# High complication rate after extendible endoprosthetic replacement of the proximal tibia: a retrospective study of 42 consecutive children

**DOI:** 10.1080/17453674.2018.1534320

**Published:** 2018-10-29

**Authors:** Panagiotis Tsagozis, Michael Parry, Robert Grimer

**Affiliations:** 1 The Royal Orthopaedic Hospital, Bristol Road South, Birmingham, UK;; 2 Department of Orthopaedic Surgery, Karolinska University Hospital and Karolinska Institute, Stockholm, Sweden

## Abstract

Background and purpose — The long-term outcome of reconstruction with extendible prostheses after resection of tumors the proximal tibia in children is unknown. We investigated the functional outcome, complication rate and final limb salvage rate after this procedure.

Patients and methods — 42 children who had a primary extendible replacement of the proximal tibia for bone tumor with a Stanmore implant between 1992 and 2013 were identified in the department’s database. All notes were reviewed to identify the oncological and functional outcomes, the incidence of complications and the rate of amputation. 20 children were alive at final follow-up. Median follow-up time was 6 years and minimum follow-up for surviving patients was 3 years.

Results — The overall limb salvage rate was 35/42; amputation was needed in 7 children. 15 implants were revised with a new implant. The Musculoskeletal Tumor Society Score was 73% (40–93) at final follow-up. The overall complication rate was 32/42. Soft tissue problems were the most common mode of complication, noted in 15 children, whereas structural failure and infection occurred in 12 children each. Use of prostheses with non-invasive lengthening was associated with a higher infection rate as compared with conventional ones (4/6 vs. 8/36) and inferior limb survival.

Interpretation — Extendible replacements of the proximal tibia allow for limb salvage and satisfactory late functional outcome but have a high rate of complications. The use of non-invasive lengthening implants has not shown any benefit compared with conventional designs and is, rather, associated with higher risk for infection and amputation.

Extendible (growing) prostheses for surgical management of children with tumors of the lower extremity have been used since the mid-1970s. Initial designs allowed for extension through the replacement of modular parts, and later through the use of a screw-based extension mechanism (Henderson et al. 2012, Ruggieri et al. 2013, Albergo et al. 2017). More recently, non-invasive lengthening models have been introduced that rely on the presence of an external electromagnetic field that controls an internal motor or a spring mechanism for elongation (Gupta et al. 2006, Gilg et al. 2016).

The use of extendible prostheses for the reconstruction of the proximal tibia has not been described in detail. The proximal tibia is the second most common site for primary skeletal sarcomas and contributes half of the overall growth of the tibia, and one-quarter of the growth of the lower limb. The ability to maintain length of this segment is important following tumor excision (Albergo et al. 2017). Early-generation implants and reconstruction of the extensor mechanism with a mesh, which is thought to predispose to infection, carried a high complication risk (Grimer et al. 2000).

We investigated the outcome after primary reconstruction of the proximal tibia with modern, commercially available extendible endoprostheses in 42 children, using muscle flap reconstruction of the extensor mechanism in all children and non-invasive implants in 6 children.

## Patients and methods

This is a retrospective study of 42 consecutive children operated at the Royal Orthopaedic Hospital (Bristol Road South, Birmingham, UK) between 1992 and 2013 with extendible proximal tibia endoprosthesis for bone tumor. The implants were manufactured by Stanmore (Stanmore Implants Worldwide Ltd, Elstree, UK).

Children were identified from the institution’s prospectively maintained database. All notes were reviewed. Children who had had previous surgery in the area (except for biopsy) were not included.

There were 23 girls and 19 boys with a mean age of 10 years (6–13). The most common diagnosis was osteosarcoma (35 children) followed by Ewing’s sarcoma in 5, and other primary bone sarcomas in 2. Mean tumor size was 9 cm (5–17). Lung metastases at presentation were seen in 2 children, bone metastases in 1, and combined bone and lung metastases in another 2.

All implants were custom-made. 36 used minimally invasive lengthening by a modified Allen key and 6 were non-invasive designs, relying on an external drive unit that generates a rotating electromagnetic field. Mean implant length was 17 cm (8–24) and similar between minimally invasive (mean length 17 cm) and non-invasive (mean length 16 cm) implants. 4 implants were surface treated with silver (Agluna, Accentus Medical, Oxford, UK). Implants were cemented into the intramedullary cavity of the remaining tibia, whereas the stem inserted in the proximal femur was uncemented in order to allow growth of the distal femoral physis. Surgical margins were clear in 41 children and there was a microscopically positive margin in 1 child. Reconstruction of the extensor mechanism and coverage of the fascial defect was done using a medial gastrocnemius flap. Antibiotic prophylaxis varied: intravenous cephalosporins were used in the first decade while a combination of flucloxacillin with gentamycin for 24 hours is the current regimen. Thromboembolism prophylaxis consisted of pneumatic compression devices on the contralateral leg as well as thromboembolic deterrent stockings. Children were allowed to fully weight-bear immediately. Mobilization was routinely done without the use of any brace or cast.

Complications were described according to the system adopted by the International Society of Limb Salvage (ISOLS), and survival analysis was done according to the Kaplan–Meier method (Henderson et al. 2014). Functional outcomes were recorded using the modified scoring system of the Musculoskeletal Tumor Society (MSTS) (Enneking et al. 1993). Leg-length discrepancy was measured on calibrated whole-length radiographs of both extremities. There was no blinding in the evaluation of data. Oncological follow-up was standardized according to the European Society for Medical Oncology guidelines with clinical examination and chest radiographs every 3 months for the first 2 years, every 6 months up to the 5th year after surgery, and then annually for another 5 years. Implants were controlled for another 2 years with annual radiographs. Median follow-up time was 6 years and minimum follow-up for surviving patients was 3 years. Patients who had received amputation were included in the survival analysis.

### Statistics

Statistical analysis was performed using SPSS (version 20, IBM Corp, Armonk, NY, USA) and STATA (version 13) software (StataCorp LLC, College Station, TX, USA). Survival curves were prepared according to the Kaplan–Meier method, and compared using the log-rank and modified Wilcoxon–Breslow test. Competing risk analysis was done using the method of Pepe and Mori. Analysis of possible prognostic factors was performed using the Cox proportional hazard test. Pearson’s chi-square (χ2) test was used for comparisons between groups. All tests were double-sided, and a p-value of ≤0.05 was considered significant. In parentheses, we present range of values unless otherwise stated. When 95% confidence intervals are shown in parentheses they are stated as CI. The core facility of the Statistics Department of the Karolinska Institute was consulted for data analysis.

### Ethics, funding, and potential conflicts of interest

The work is a retrospective study that complies with Institutional Guidelines. No funding was received for this work. The authors declare no conflict of interest.  

## Results

### Oncological outcome

**Figure 1:**
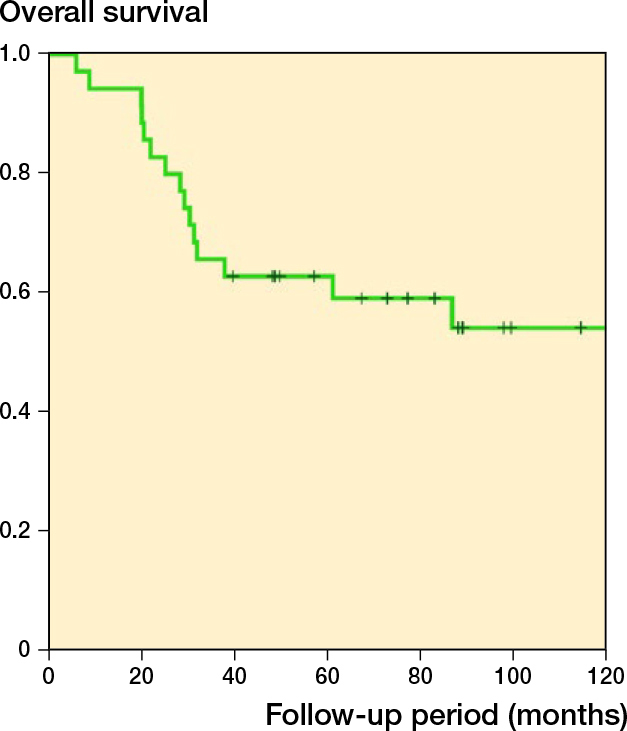
Kaplan–Meier analysis for overall survival of 42 children who underwent proximal tibial replacement for bone tumors.

Of the 42 children, 20 were alive at final follow-up. Overall survival (OS) was 63% (CI 47–79) at 5 years and 53% (CI 36–70) at 10 years (Figure 1). OS was not affected by tumor type. Local recurrence occurred in 2 children.

### Functional outcome

Mean MSTS score was 57% (30–87) at 1 year and 73% (40–93) at final follow-up. Mean range of movement was 72° (15–130) at 1 year and 89° (25–120) at final follow-up. The mean extension lag at 1 year was 32° (0–90), diminishing to 17° (0–50) at final follow-up. Radiographs allowing final leg length measurement were available in 25 children and mean tibia lengthening was 1.5 cm (0–7). In 12/25 of cases tibia lengthening was less than 0.5 cm, and in 10 of these this was attributed to local complications including infection and other soft-tissue problems (wound complications, stiffness, pain, joint contraction) that often resulted in prolonged treatment and secondary operations that impeded lengthening, whereas mechanical failure of the prosthesis was responsible for the remaining 2 cases of non-lengthening. The final average leg length difference at final follow-up was 1.8 cm (0–6).

**Table:** Univariate Cox regression analysis of different complication types on prosthesis survival (PS) as well as limb survival (LS) of 42 children who underwent proximal tibial replacement for bone tumors (hazards ratio with 95% confidence intervals)

Type of complication	Number of complications	Hazard ratio PS (CI)	Hazard ratio LS (CI)
I (soft tissue)	15	3 (1–9)	6 (1–37)
II (loosening)	1	0.6 (0.1–4.8)	1 (0–1000)
III (structural)	12	0.7 (0.2–2.1)	0.2 (0.1–1.6)
IV (infectious)	12	3.5 (1.1–10)	14 (2–106)
V (recurrence)	2	1.1 (0.3–5.0)	2.3 (0.3–19)

### Complications

Complications of any kind were observed in 32/42 children. There was no correlation between the age of the patient and the incidence of complications.

**Type I** complications (soft tissue problems) arose in 15 children. Although intensive physiotherapy was offered to all children, 9 of them eventually developed persistent stiffness and underwent 1 or more manipulations of the joint under general anesthesia. 4 children experienced wound dehiscence and/or necrosis. 1 patient underwent Trevira tube augmentation of the extensor mechanism due to persistent extensor deficit and 1 had excision of scar tissue and correction of a fixed flexion deformity with revision of the implant at a later time-point.

**Type II** failure (symptomatic aseptic loosening) was documented in 1 child, who had his implant revised for this reason 9 years after insertion.

**Type III** (structural) failure occurred in 12 children. Failures of the expansion mechanism of the prosthesis were seen in 7 children, 5 of whom were revised to another growing prosthesis; the remaining 2 refused reoperation. Periprosthetic fractures occurred in 4 children. These were managed by cast treatment in 1, open reduction and internal fixation in 1, and revision of the prosthesis in 1, while amputation was required in 1 patient due to poor bone quality and the presence of severe soft-tissue contractions. The anti-rotation lug failed in 1 child, whose implant was also revised.

**Type IV** failure (infection) was seen in 12 children. Of these, 5 eventually underwent amputation, as the infection could not be controlled. 4 children underwent 2-stage revision, 1 of them twice. None of these 4 children ended in amputation. 2 children had a single-stage revision for infection, 1 being successfully managed and 1 ending in amputation. 6 children developed infections that were managed with antibiotics and soft-tissue procedures, but 4 of them eventually required amputation as the infection could not be controlled. There was a higher incidence of infection in the case of non-invasive designs (4/6 vs. 8/36, p = 0.05). The incidence of infection was similar between silver-coated implants and those not silver coated (11/36 vs. 1/6, p = 1). There was no difference either between prophylactic antibiotic regimens used.

**Type V** failure (local recurrence) was documented in 2 children. Of these, 1 was treated by amputation, the other by local excision and adjuvant radiotherapy.

**Type VI** failure (growth arrest, joint deformity) was noted in 1 of the children, who had a 6 cm limb shortening due to growth arrest at the level of the distal femoral growth plate.

### Prosthesis survival, limb salvage rate, and use of non-invasive implants

**Figure 2:**
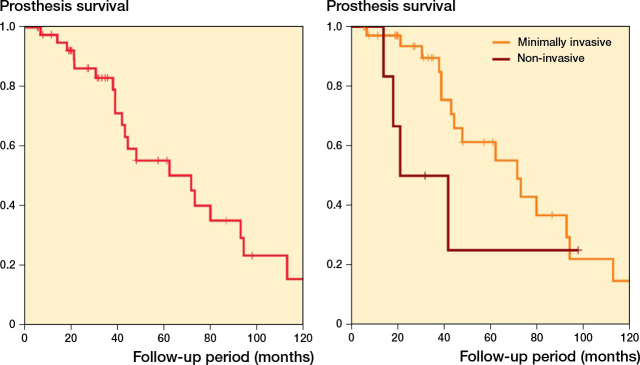
(left panel) Kaplan–Meier analysis for prosthesis survival of 42 children who underwent proximal tibial replacement for bone tumors. (right panel) Minimally invasive implants had a marginally superior survival as compared with non-invasive ones (p = 0.02 according to the modified Wilcoxon method and p = 0.2 according to the log-rank test, showing that minimally invasive implants have better survival only in the early follow-up period).

Prosthesis survival was 55% (CI 37–73) at 5 years and 25% (CI 7–43) at 10 years (Figure 2). 22/42 implants failed due to revision or amputation of the limb. The impact of the different types of complication on prosthesis survival is shown in the Table. Infection was the sole factor predisposing to inferior prosthesis survival in univariate analysis. Silver-coated implants were not associated with superior prosthesis survival (p = 0.8). There was a marginal difference regarding prosthesis survival among minimally invasive and non-invasive implants, with the latter showing a higher failure rate in the early follow-up period (Figure 2). This association was not observed in a competing risk survival analysis with death as a competing factor (Figure 3, Supplementary data).

**Figure 4:**
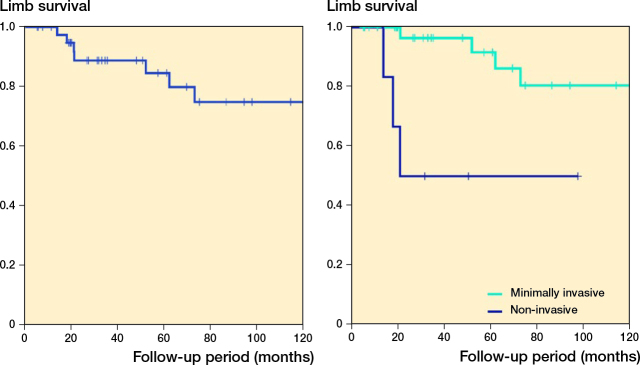
(left panel) Limb survival according to Kaplan–Meier, for 42 children who underwent proximal tibial replacement for bone tumors. (right panel) Minimally invasive implants were associated with superior limb survival (p = 0.003 according to the log-rank test and p < 0.001 according to the modified Wilcoxon method).

Amputation was performed in 7 children. Kaplan–Meier analysis showed a limb survival of 85% (CI 72–98) at 5 years and 75% (CI 58–92) at 10 years (Figure 4). Infection was the leading cause of amputation (5 children), whilst local recurrence accounted for 1 amputation and 1 was attributed to non-healing fracture. The development of a postoperative infection was associated with significantly inferior limb survival (p = 0.01). Other type of complications did not have a significant effect on limb survival in our series (Table). Furthermore, possible confounding factors such as sex (p = 0.7 for prosthesis survival and p = 0.8 for limb survival) and age (p = 0.3 for prosthesis survival and p = 0.3 for limb survival) had no statistically significant effect.

There was a higher incidence of infection in the case of non-invasive designs (4/6 vs. 8/36, p = 0.05). The higher infection rate observed in children with non-invasive implants was reflected in a higher risk of requiring a secondary amputation. Use of minimally invasive implants was associated with a superior limb salvage rate as compared with non-invasive ones (Figure 4), even though this correlation did not reach statistical significance in a competing risk analysis with death as a competing factor (Figure 5, Supplementary data).

## Discussion

Limb salvage is the preferred mode of treatment for malignant tumors of the extremities, since it results in superior functional outcome, offering at the same time equivalent local control and overall survival compared with amputation (Simon et al. 1986, Rougraff et al. 1994). However, management of the growing individual poses challenges: the skeletal growth must be taken into consideration when planning reconstruction. The use of extendible prostheses has gained popularity as it allows immediate mobilization and improved cosmesis for the patient (Henderson et al. 2012, Picardo et al. 2012, Schinhan et al. 2015). For tumors arising in the proximal tibia, however, concerns are that poor soft tissue coverage may increase the risk of infection and stiffness, as well as difficulty in reconstruction of the extensor mechanism resulting in restriction in the range of movement (Albergo et al. 2017). Previous study in a limited number of children, who were not included in this report, highlighted the high risk for complications after extendible endoprosthetic reconstruction for proximal tibia tumors (Grimer et al. 2000). However, this option is chosen for many children as it allows for retaining a cosmetically near-normal limb, which is not the case for amputation or rotationplasty. On the other hand, rotationplasty (but not an above-knee amputation) allows for an active lifestyle including sport activities, which are not compatible with endoprosthetic reconstruction due to the risk for mechanical failures. We present our experience from the largest series of patients in the medical literature, showing that limb salvage is feasible but at the expense of a high complication rate.

This study has a number of limitations. First, it is a retrospective case series: prospective randomized studies are not possible given the rarity of diagnosis. Second, for the same reason, the study expands over 3 decades. However, all operations were done with similar implants from a single manufacturer, following a common surgical technique and postoperative aftercare. Follow-up was also standardized.

The functional outcome, as judged by the MSTS score, was satisfactory at final follow-up, although it was rather poor at 1 year after surgery. The prostheses were also capable of retaining a good range of movement of the knee joint. Leg-length discrepancy was mainly due to inability to lengthen the prosthesis, often due to local complications such as soft-tissue problems and infection requiring secondary operations and prolonged medical treatment, rather than mechanical failure or femoral growth disturbance. We found a high rate of complications in approximately 3 out of 4 children. Although soft-tissue problems were the most common type of complication, and often required repeated operations and resulted in a poor functional outcome, they do not usually pose a threat to the limb. On the other hand, infection remains the main concern, encountered in almost one-third of children, and was the main threat for amputation. The poor soft tissue envelope of the anterior tibia predisposes to the high rate of infection, which is high even when a gastrocnemius flap is used for soft-tissue reconstruction. Silver coating did not have a protective effect in our study, in contrast to previous data, which is probably attributable to the low number of patients in this report (Hardes et al. 2010, Wafa et al. 2015). Once an infection developed, the chance of it being controlled with either a 1-stage or 2-stage revision was 0.8. For those treated with more conservative procedures or suppressive antibiotics, the risk of requiring an amputation to control the infection rose to 0.6. We also observed a high risk for structural failures, the commonest one being inability to lengthen the prosthesis or maintain lengthening due to faults in the extension mechanism. Other studies have also documented a high incidence of mechanical failures with expandable prostheses, highlighting the need for improvement in the design and manufacture of the implants (Eckardt et al. 1993, Ruggieri et al. 2013, Cipriano et al. 2015).

We observed an increased rate of infection with a higher risk for amputation in the case of non-invasive implants, although the number of non-invasive implants in our series is too small to draw safe conclusions. Non-invasive implants are bulkier in order to accommodate the lengthening mechanism, which may explain their higher risk of infection. Size of the implant is probably the most critical factor regarding susceptibility to infection, as opposed to other sites with superior soft tissue coverage where non-invasive implants have shown low risk for type IV failure (Picardo et al. 2012, Schinhan et al. 2015, Gilg et al. 2016). We do not feel that antibiotic prophylaxis, which was the same as in any megaprosthetic reconstruction, was the main factor that contributed to the high infection rate. Advances in implant design, offering smaller implants, may decrease the risk for complications. Moreover, we believe that avoidance of soft-tissue tension during wound closure is crucial for avoiding infection.

The prosthesis survival curve illustrates the fact that in most cases the initial implant is eventually revised. Although the prostheses used in our study are manufactured to be able to function as the final implant, in practice they are often revised to an adult-type implant at some stage.

In summary, our results suggest that extendible endoprosthetic reconstruction of the tibia allows for limb preservation in most children but is unfortunately offset by a high risk of complications. In the majority of cases this risk is acceptable to the children and their families, but the risk for secondary amputation must be explained. Use of non-invasive prostheses has not resulted in a reduction of infections, contrary to surgeons’ expectations. Further developments in implant design are required to improve outcome, reducing the risk of both mechanical and infective complications. Other methods might also be considered, such as biological reconstruction or the use of conventional implants combined with epiphysiodesis in children near adolescence, as well as rotationplasty or amputation.

### Supplementary data

Figures 3 and 5 are available as supplementary data in the online version of this article, http://dx.doi.org/ 10.1080/17453674.2018.1534320

Study conception and design: RG. Data analysis: PT. Manuscript preparation. PT, MP, RG

*Acta* thanks Gerard Schaap and Reinhard Windhager for help with peer review of this study.

## Supplementary Material

Supplemental Material
