# Supplementary material for: High complication rate after extendible endoprosthetic replacement of the proximal tibia: a retrospective study of 42 consecutive children
Source: Acta Orthop. 2018 Oct 29;89(6):678–82. doi: 10.1080/17453674.2018.1534320 (PMC6300744; doi:10.1080/17453674.2018.1534320)
Supplement: Supplemental Material [file IORT_A_1534320_SM9746.pdf]

Supplementary data

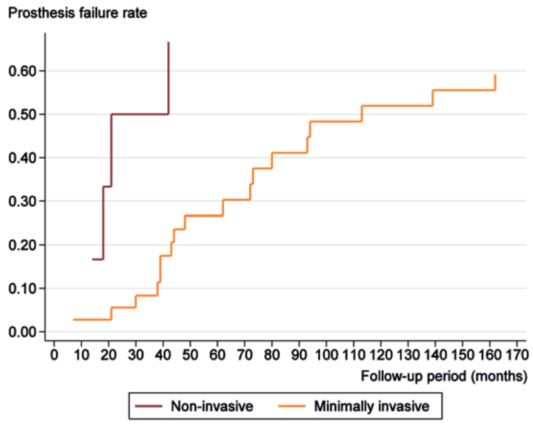

Figure 3. Competing risks survival analysis regarding prosthesis survival of 42 children who underwent proximal tibial replacement for bone tumors, with death as the competing factor. Minimally invasive implants had similar survival to non-invasive ones (p = 0.2).

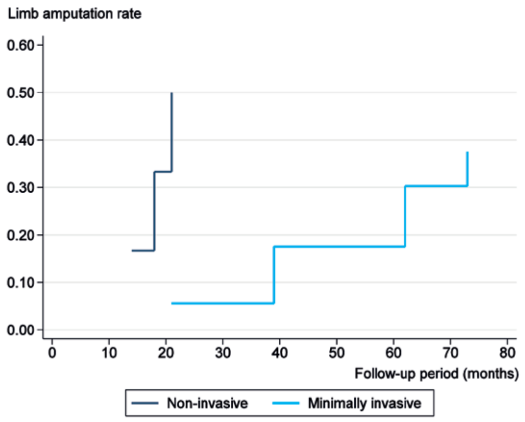

Figure 5. Competing risks survival analysis regarding limb survival of 42 children who underwent proximal tibial replacement for bone tumors, with death as the competing factor. Minimally invasive implants had similar survival to non-invasive ones (p = 0.1).
